# Transcriptional changes detected in fecal RNA of neonatal dairy calves undergoing a mild diarrhea are associated with inflammatory biomarkers

**DOI:** 10.1371/journal.pone.0191599

**Published:** 2018-01-26

**Authors:** Fernanda Rosa, Sebastiano Busato, Fatima C. Avaroma, Kali Linville, Erminio Trevisi, Johan S. Osorio, Massimo Bionaz

**Affiliations:** 1 Department of Animal and Rangeland Sciences, Oregon State University, Corvallis, Oregon, United States of America; 2 Escuela Agrícola Panamericana El Zamorano, El Zamorano, Francisco Morazán, Honduras; 3 Department of Dairy and Food Sciences, South Dakota State University, Brookings, South Dakota, United States of America; 4 Istituto di Zootecnica, Facoltà di Scienze Agrarie, Alimentari e Ambientali, Università Cattolica del Sacro Cuore, Piacenza, Italy; University of Illinois, UNITED STATES

## Abstract

After birth, a newborn calf has to adapt to an extrauterine life characterized by several physiological changes. In particular, maturation of the gastrointestinal tract in a new environment loaded with potential pathogens, which can predispose neonatal calves to develop diarrhea, and is a major cause of morbidity and mortality during the first 4 wks of life. We aimed to investigate the inflammatory adaptations at a transcriptomic level in the gastrointestinal (GI) tract to a mild diarrhea in neonatal dairy calves using RNA isolated from fresh fecal samples. Eight newborn Jersey male calves were used from birth to 5 wks of age and housed in individual pens. After birth, calves received 1.9 L of colostrum from their respective dams. Calves had ad-libitum access to water and starter grain (22% CP) and were fed twice daily a total of 5.6 L pasteurized whole milk. Starter intake, body weight (BW), fecal score, withers height (WH), and rectal temperature (RT) were recorded throughout the experiment. Blood samples were collected weekly for metabolic and inflammatory profiling from wk 0 to wk 5. Fresh fecal samples were collected weekly and immediately flash frozen until RNA was extracted using a Trizol-based method, and subsequently, an RT-qPCR analysis was performed. Orthogonal contrasts were used to evaluate linear or quadratic effects over time. Starter intake, BW, and WH increased over time. Fecal score was greatest (2.6 ± 0.3) during wk 2. The concentrations of IL-6, ceruloplasmin, and haptoglobin had a positive quadratic effect with maximal concentrations during wk 2, which corresponded to the maximal fecal score observed during the same time. The concentration of serum amyloid A decreased over time. The mRNA expression of the proinflammatory related genes *TLR4*, *TNFA*, *IL8*, and *IL1B* had a positive quadratic effect of time. A time effect was observed for the cell membrane sodium-dependent glucose transporter *SLC5A1*, for the major carbohydrate facilitated transporter *SLC2A2*, and water transport function *AQP3*, where *SLC5A1* and *AQP3* had a negative quadratic effect over time. Our data support the use of the fecal RNA as a noninvasive tool to investigate intestinal transcriptomic profiling of dairy calves experiencing diarrhea, which would be advantageous for future research including nutritional effects and health conditions.

## Introduction

After birth, the gastrointestinal tract (GI) of a newborn calf undergoes morphological and functional adaptations, which starts with the ingestion of colostrum affecting the gastrointestinal motility as well as the acquisition of the passive immunity [1, 2]. In addition to the gastrointestinal maturation, the calf has to adapt to a new environment, which is loaded with pathogens, thermoregulate its own body and transition from pre-ruminant to ruminant during the weaning period. Together, these factors can prone calves to develop diarrhea, which is the leading cause of mortality in neonatal dairy calves in the US during the first 4 weeks of life [3]. Fecal score is commonly used by researchers and dairy producers to keep track and identify young animals with diarrhea. Also, more sophisticated methods such as microscopy and bacterial culturing have been used to identifying specific strains of infectious pathogens causing diarrhea. Blood biomarkers of inflammation, oxidative stress, metabolism are useful indicators of overall animal health. However, these analyses along with fecal score do not provide additional information on the actual biological adaptations the GI tract undergoes during the onset of diarrhea. Epithelial cells from the GI tract act as a barrier against pathogens, and the daily shedding of these cells into feces has been used in rodents and humans as a source of RNA to evaluate gene expression in the GI tract [4]. The exfoliation of these cells has been associated with intestinal natural turnover process to preserve tissue structure [5]. This is a noninvasive method that can be used instead of performing tissue biopsies and postmortem tissue analysis [6]. Originally, RNA from fecal samples was used for detecting molecular biomarkers of colon cancer in rats [7, 8] and later for intestinal development and function in adult humans [9, 10]. The basis for using fecal RNA is that, unlike mammalian RNA, bacteria RNA lacks a polyA tail making possible to discriminate between RNA from the host and RNA from bacteria by targeting RNA with the polyA. Certain strains of fungi, which are also considered pathogens, can produce polyA tail [11, 12], however, the extent and influence of these strains in the fecal RNA remain unknown. In humans, the RNA isolated from stool samples were used to study nutritional regimens [13], as well as, to quantify intestinal gene expression profiles in developing human infants [14]. In fact, in the latter study, using fecal RNA, Chapkin and collaborators [14] were able to differentiate between neonates that were breastfed and formula-fed by their different transcriptome profile. These data underscore the potential of the fecal RNA method to study biological alterations to the GI tract product of nutritional effects. Similarly, such method could be applied to study the effects of inflammatory conditions arise from diarrhea in neonatal dairy calves.

Therefore, we hypothesized that inflammatory signals could be detected in gene expression of fecal RNA in response to adaptations prior to- and during the onset of diarrhea in neonatal calves. Thus, the objective of this study was to use RNA isolated from fecal samples to study the molecular adaptations to a mild-diarrhea via assessment of expression of specific genes involved in inflammation and cell membrane transporters through RT-qPCR and validate the findings using well-established profiling of inflammatory biomarkers in blood.

## Materials and methods

### Experimental design, animal management

The Institutional Animal Care and Use Committee (IACUC) of the Oregon State University (OSU) approved all procedures for this study (Protocol # 4747). Eight healthy newborn male Jersey calves from the OSU Dairy Center were used in the study by applying the following inclusion criteria: 1) calving difficulty score < 3, 2) single calf, and 3) calf birth weight ≥ 34 kg. After birth, calves were supplemented with BO-SE (Merck Animal Health, Germany) and vaccinated with CALF-GUARD^®^ (Bovine Rota-Coronavirus vaccine, Zoetis, MI), and received 1.9 L of colostrum from the respective dam in the first two feedings. Calves remained in the study from birth to 5 wk of age (i.e., preweaned period). Calves were housed in individual pens bedded with straw at OSU Dairy Center and fed twice daily with pasteurized whole cow milk. Calves had ad libitum access to water and a starter grain (Ampli-Calf^®^; 22% CP, Purina Animal Nutrition, Shoreview, MN) throughout the experiment.

Growth performance including BW and withers height (WH) were recorded weekly before morning feeding. Health evaluations including fecal score [scale 1–4, 1: firm, well formed (not hard); 2: soft, pudding-like; 3: runny, pancake batter; 4: liquid, splatters; [15]] and respiratory score [scale 1–5, 1: normal 2: runny nose, 3: heavy breathing, 4: cough moist, 5: cough dry; [15]] were recorded daily throughout the experiment. Based on the above fecal score scale, we classified calves with a mild-diarrhea with fecal score of 3, and diarrhea with fecal score of 4. Rectal temperature was recorded daily until 21 d of age. Fresh fecal samples (~ 10 g) were collected from the rectum weekly ~30 min after morning feeding from wk 1 to 5 (or 7 to 35 d). Fecal samples were immediately flash-frozen in liquid N_2_ and kept at -80°C until further analysis.

### Blood sample collection

Blood samples were collected prior to morning feeding from the jugular vein using a 20-gauge BD vacutainer needles (Becton Dickinson, Franklin Lakes, NJ). Samples were collected into evacuated tubes (10 mL, BD Vacutainer^®^, Becton Dickinson, Franklin Lakes, NJ) containing either serum clot activator or sodium heparin within 30 minutes after the morning feeding starting from the 1 d after calving (i.e., 0 wk) and subsequent samples were collected weekly until wk 5. After blood collection, tubes with sodium heparin were placed on ice (4°C), and tubes with clot activator were kept at 21°C (~30 min) until centrifugation. Serum and plasma were obtained by centrifugation of clot activator and sodium heparin tubes, respectively, at 1,200 × *g* for 15 minutes. Aliquots of serum and plasma were frozen (-80°C) until further analysis.

### Blood biomarkers

Blood samples were analyzed for energy metabolites [i.e., glucose, non-esterified fatty acids (NEFA), and β-hydroxybutyric acid (BHB)], muscle mass catabolism (i.e., urea and creatinine), liver function [i.e., albumin, cholesterol, total bilirubin,gamma-glutamyl transferase (GGT), paraoxonase (PON), glutamic-oxaloacetic transaminase (GOT)], inflammation [i.e., Serm Amyloid A (SAA), haptoglobin, ceruloplasmin, Interleukin (IL)-6, and IL-1β], and oxidative stress [i.e., myeloperoxidase, reactive oxygen metabolites (ROM), ferric reducing antioxidant power (FRAP)] using kits purchased from Instrumentation Laboratory (Lexington, MA) following the procedures described previously [16, 17].

### RNA isolation, cDNA synthesis, primer design and evaluation and quantitative PCR

Complete details regarding the RNA isolation and cDNA synthesis are presented in S1 File. The RNA isolation from fecal samples was carried using the RNeasy Plus Mini Kit (Qiagen, Cat. No. 74134), following the manufacturer’s instructions with some modifications. Briefly, prior to the RNA isolation, the fecal samples (~200 mg) were homogenized using a Bullet Blender Next Advance (Laboratory Instruments, USA) immersed in 1.2 mL of TRIzol^®^ Reagent (Cat# 15596018, Ambion, Carlsbad, CA, USA). After homogenization, 240 μl of chloroform (EMD Millipore, Germany, Cat. No. CX1055-6) was added in order to isolate the RNA from the organic phase. The RNA quantity (822.9 ± 491.8 ng/μL; mean ± SD) and purity as 260/280 ratio (1.98 ± 0.3) were determined via spectrophotometry using SpectraMax^®^ Plus 384 (Molecular Devices, Sunnyvale, CA, USA). The RNA integrity was assessed using the TapeStation^®^ (Agilent Technologies), and the final RIN number was 3.4 ± 1.5 with a range from 1.8 to 7.4. This RIN value can be considered as a limitation for this study since is lower than desirable values (≥ 5) for RT-qPCR. However, in this study, this effect did not translate in an adverse effect on the PCR amplification curves across samples. Future optimizations to this technique will improve this parameter.

The complementary DNA (cDNA) synthesis was performed according to Bionaz and Loor [18] with some modifications. Each cDNA was synthesized by reverse transcription using 500 ng RNA, 1 μL Random Primers (Cat# 48190–011, Invitrogen, Carlsbad, CA, USA), and 5 μL DNase/RNase-free water (Cat# 10977–015, Life Technologies, Grand Island, NY). The mixture was incubated at 65°C for 5 min and kept on ice for 3 min. A total of 9 μL of Master Mix composed of 1 μg dT18 (Invitrogen, Carlsbad, CA, USA), 2 μL 10 mM dNTP mix (Cat# 18427–013, Invitrogen, Carlsbad, CA, USA), 4 μL 5 × Reaction Buffer, 0.25 μL RevertAid Transcriptase (200 U/μL; Thermofisher; Cat. No. EP0441), 0.125 μL Rnase Inhibitor (40U/μL; Thermofisher; Cat. No. EO0382), and 1.625 μL DNase/RNase-free water were added to each sample. The second step of the cDNA synthesis reaction was performed as follow: 25°C for 5 min, 42°C for 60 min, and 70°C for 5 min. The synthesized cDNA was then diluted 1:3 with DNase/RNase-free water. The qPCR reaction was performed in a 7900HT Fast Real-Time PCR System (Applied Biosystems, USA) in MicroAmp^®^ Optical 384-well Reaction Plate (Applied Biosystems, USA) as described in Bionaz and Loor [18]. The qPCR efficiency and quantification cycle values were obtained for each reaction using LinRegPCR [19]. The genes glyceraldehyde 3-phosphate dehydrogenase (*GAPDH*), ribosomal protein S15a (*RPS15A*) and Peptidylprolyl isomerase A (*PPIA*), β-actin (*ACTB*), β-2-microglobulin (*B2M*), and ribosomal protein 9 (*RPS9*) were used as internal control genes (ICG). With the exception of *PPIA*, which was designed and sequenced in this study (S1 Table), all other ICGs were obtained from Kadegowda et al. [20]. The geometric mean of the internal control genes was used to normalize the expression. The stability of the normalization factor was assessed using geNorm software [21] with a favorable final pairwise variation of 0.19.

The target genes selected (Table 1) to be evaluated in fecal RNA samples were related to the inflammatory response (i.e., *TLR2*, *TLR4*, *NFKB1*, *TNFA*, *IL1B*, *IL8*, and *IFNG*) and cell membrane transporters (i.e., *SLC5A1*, *AQP3*, and *SLC2A2*). The primers for *SLC5A1*, *AQP3*, and *SLC2A2* were designed and tested as previously described by Bionaz and Loor [18] (S1 Table). All amplicon sequences were confirmed using the National Center for Biotechnology Information Database (NCBI; S2 Table).

**Table 1:** Target genes and their biological function.

Symbol	Name	Biological function and process	Source
*Inflammatory response*			
*TLR2*	Toll-like receptor 4	Participates in the innate immune response to gram positive microbial agents	[16]
*TLR4*	Toll-like receptor 2	Participates in the innate immune response to gram negative microbial agents	[16]
*NFKB1*	Nuclear factor of kappa light polypeptide gene enhancer in B-cells 1	It is involved in many biological processes such as inflammation, immunity, differentiation, cell growth, tumorigenesis, and apoptosis	[16]
*TNFA*	Tumor necrosis factor-α	Involved in the regulation of a wide spectrum of biological processes including cell proliferation, differentiation, apoptosis, lipid metabolism, and coagulation	[16]
*IL1B*	Interleukin 1,β	Main functions include mediator of the inflammatory response, and is involved in a variety of cellular activities, including cell proliferation, differentiation, and apoptosis	[16]
*IL8*	Interleukin 8	Chemokine protein with major functions as mediator of inflammatory response and chemoattractant for immune cells	[22]
*IFNG*	Interferon, gamma	Important antiviral activity and immunoregulatory functions	[23]
*Cell membrane*			
*SLC5A1*	Solute carrier family 5 member A1	Cell membrane with a sodium-glucose transporter function	This manuscript
*AQP3*	Aquaporin 3	Cell membrane with a water channel function	This manuscript
*SLC2A2*	Solute carrier family 2 member 2	The encoded protein mediates facilitated bidirectional glucose transport. Because of its low affinity for glucose, it has been suggested as a glucose sensor	This manuscript

### Statistical analysis

Data were analyzed using the PROC MIXED procedure of SAS 9.4 (SAS Institute, Inc., Cary, NC, USA). The model included time as fixed effect and calf as random. The autoregressive (1) covariance structure was used for repeated measures for all parameters analyzed. Blood metabolites and gene expression results were log-scale transformed if needed, in order to comply with a normal distribution of residuals. Daily fecal scores and starter intakes were average weekly before statistical analysis. Orthogonal contrasts were used to determine linear and quadratic effects when a trend (*P* < 0.10) for a significant time effect was observed. Statistical significance and tendencies were declared at *P* ≤ 0.05 and 0.05 ≤ *P* ≤ 0.10, respectively.

## Results

### Growth performance and health

**Fig 1:**
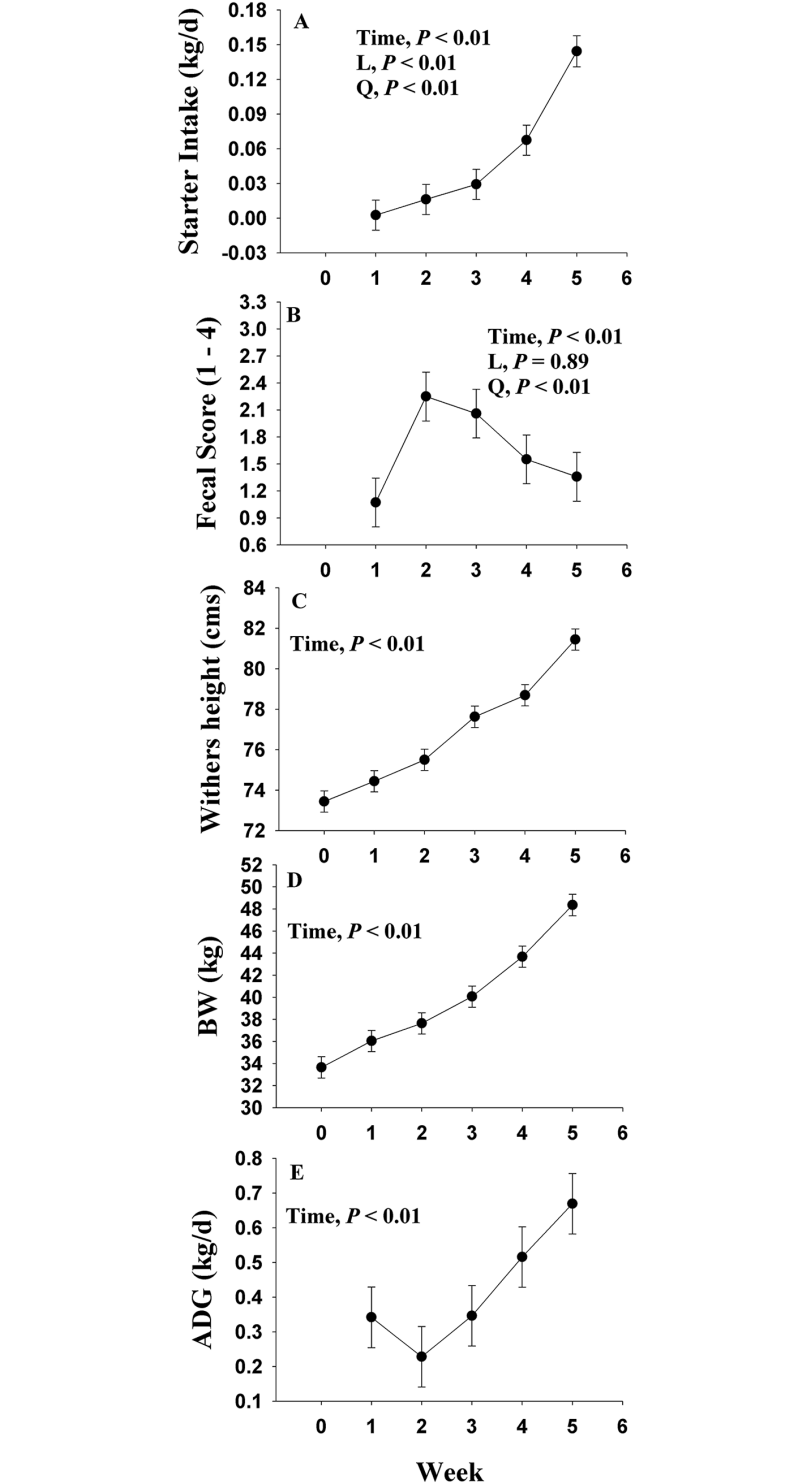
Performance parameters during the neonatal period of Jersey calves. Starter intake (A), fecal scores (B), withers height (C), body weight (D), and average daily gain (ADG) (E). The *P* values for main effect of time are shown in each plot. The time effect (*P*< 0.10) was further analyzed in starter intake and fecal scores through orthogonal contrast in order to determine linear (L) and quadratic (Q) effects over time. Values are means, with standard errors represented by vertical bars.

Main effect of time for performance parameters are shown in Fig 1. There was a time effect (*P* < 0.01) observed on starter intake, which increased over time. Similar to starter intake, BW and WH increased (*P* < 0.01) over time. Fecal score was affected by time (*P* < 0.001), where a maximal fecal score (2.6 ± 0.3) was observed during wk 2, and returned to initial values by wk 5. Based on our classification of mild-diarrhea or fecal score 3 (i.e., runny or package batter), 7 out of the 8 calves experienced at least a mild-diarrhea for at least 1 day. Similarly, 4 out of the 8 calves experience diarrhea or fecal score 4 for at least 1 day. The descriptive distribution of daily fecal scores per week presented in S1 Fig. indicates a total occurrence of ~40% and 30% for at least a mild-diarrhea events, respectively, during wk 2. The morbidity based on fecal scores was 4.6 ± 2.5 and 5.8 ± 4.3 d, for mild-diarrhea and diarrhea, respectively. Consequently, morbidity increased to 7.9 ± 5.9 d, when evaluating numbers of days calves experience at least a mild-diarrhea (i.e., fecal score ≥ 3). The maximal fecal score during wk 2 was reflected in a morbidity of 4.0 ± 3.5 d based on calves having at least a mild-diarrhea.

### Blood biomarkers

**Fig 2:**
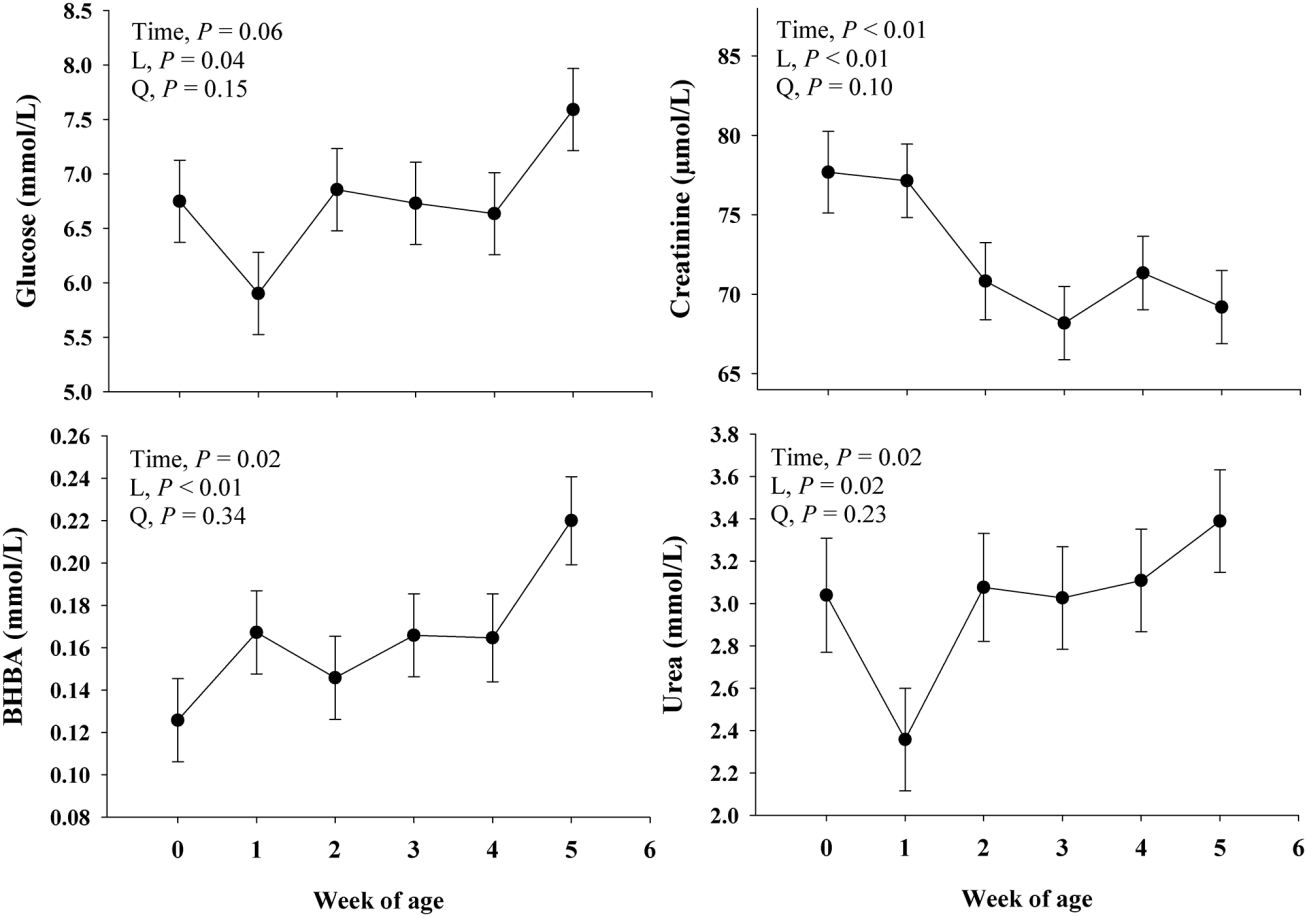
Profiles of energy (Glucose and BHBA) and muscle mass catabolism (Creatinine and urea) metabolites in blood during the neonatal period in Jersey dairy calves. The time effect (*P*< 0.10) was further analyzed through orthogonal contrast in order to determine linear (L) and quadratic (Q) effects over time. Values are means, with standard errors represented by vertical bars.

Main effect of time on energy and muscle mass catabolism metabolites are presented in Fig 2.

#### Energy metabolites

There was a time effect (*P* = 0.02) observed for BHBA and a trend (*P* = 0.06) for glucose. A linear (*P* ≤ 0.04) response was observed for glucose and BHBA, where both increased over time. The blood NEFA concentration did not change (*P* = 0.21) over time.

#### Muscle mass metabolites

A time effect (*P* ≤ 0.02) was observed for urea and creatinine. The time effect observed in urea and creatinine was partially explained by a linear response (*P* ≤ 0.02; Fig 2), where urea increased while creatinine decreased over time.

#### Liver function

**Fig 3:**
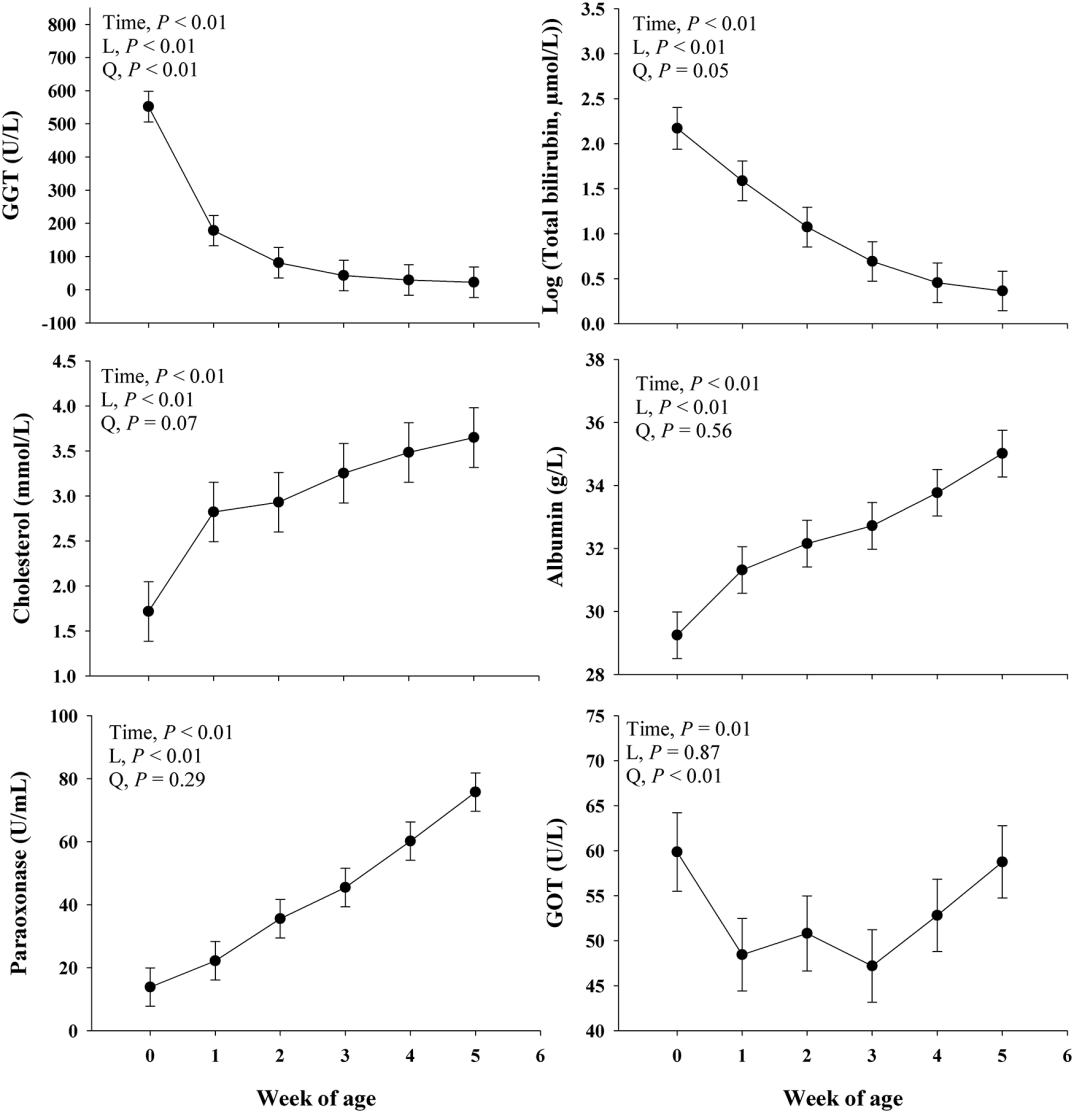
Profiles of liver function [albumin, cholesterol, total bilirubin, gamma-glutamyl transferase (GGT), paraoxonase (PON), glutamic-oxaloacetic transaminase (GOT)] metabolites in blood during the neonatal period of Jersey dairy calves. The time effect (*P*< 0.10) were further analyzed through orthogonal contrast in order to determine linear (L) and quadratic (Q) effects over time. Values are means, with standard errors represented by vertical bars.

Main effect of time on liver function biomarkers are presented in Fig 3. All of the liver function biomarkers had a significant (*P* ≤ 0.01) time effect. Albumin, cholesterol, PON, total bilirubin, and GGT had a significant (*P* < 0.01) linear response, where albumin, cholesterol, and PON increase over time, whereas GGT and total bilirubin decreased. With the exception of albumin and PON, all other biomarkers had a quadratic effect (*P* ≤ 0.05) over time, as well as a trend (*P* = 0.07) was observed for cholesterol. The GOT was the only liver function biomarker without a linear effect (*P* = 0.87) but a quadratic effect (*P* < 0.01) over time (Fig 3).

#### Inflammation

**Fig 4:**
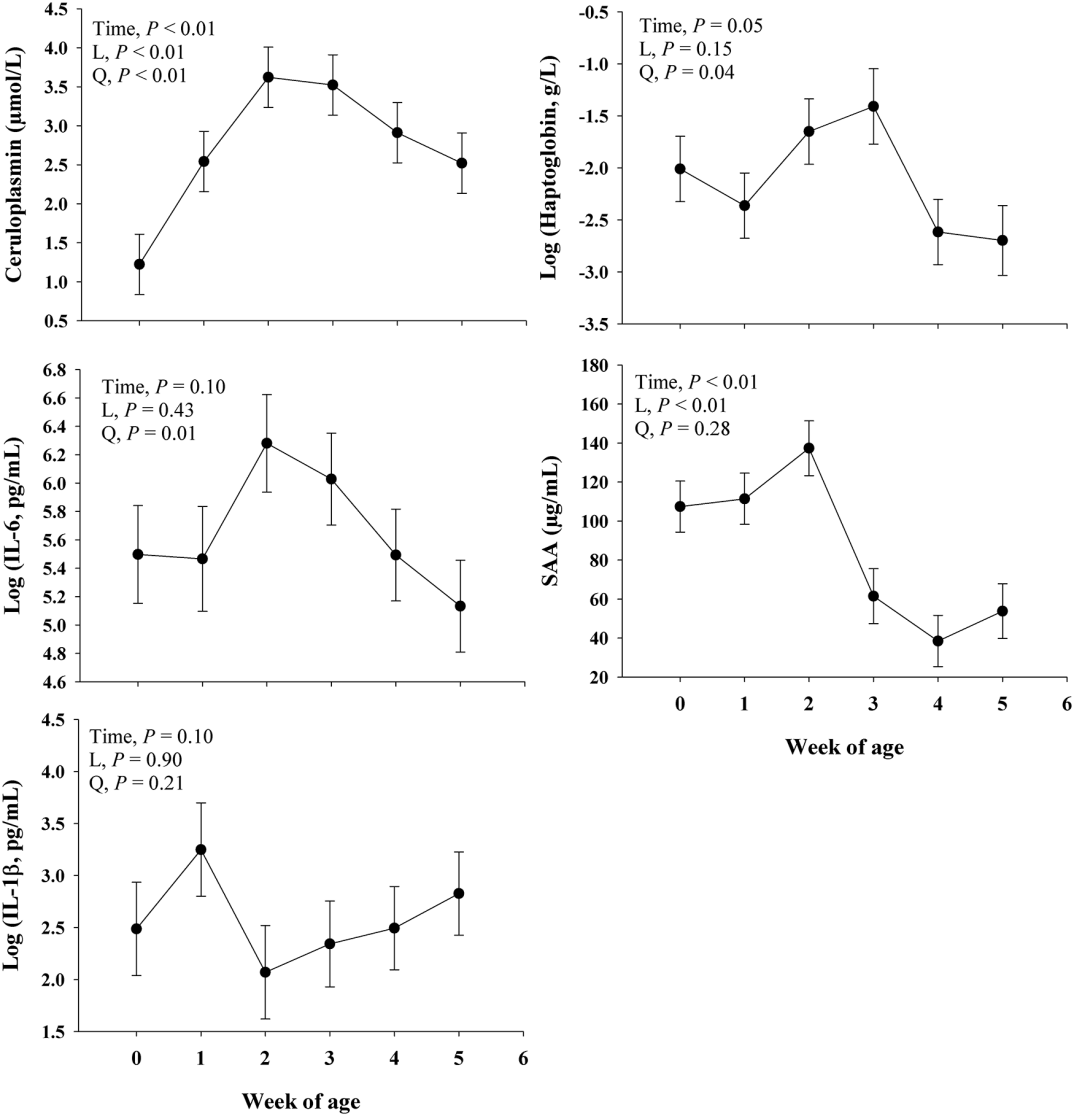
Profiles of inflammatory response (haptoglobin, ceruloplasmin, IL-6, SAA, and IL-1β) biomarkers in blood during the neonatal period in Jersey dairy calves. The time effect (*P*< 0.10) were further analyzed through orthogonal contrast in order to determine linear (L) and quadratic (Q) effects over time. An overall trend for an increase (*P* ≤ 0.08) in IL-1β at wk 1 in comparison to wk 0, 2, 3, and 4 was denoted by an asterisk (*). Values are means, with standard errors represented by vertical bars.

Main effect of time on inflammatory biomarkers are presented in Fig 4. There was a time effect (*P* ≤ 0.05) on ceruloplasmin, haptoglobin, and SAA and a trend (*P* = 0.10) on IL-6 and IL-1β over time.There was a linear response (*P* < 0.01) on ceruloplasmin and SAA, where ceruloplasmin increased over time and SAA decreased. A positive quadratic effect (*P* ≤ 0.04) was observed in ceruloplasmin, haptoglobin, and IL-6. The trend (*P* = 0.10) for a time effect on IL-1β was not explained by a linear (*P* = 0.21) or quadratic effect (*P* = 0.90), but rather by an overall trend (*P* ≤ 0.08) for an increase at wk 1 in comparison with wk 0, 2, 3, and 4 (Fig 4).

#### Oxidative stress

**Fig 5:**
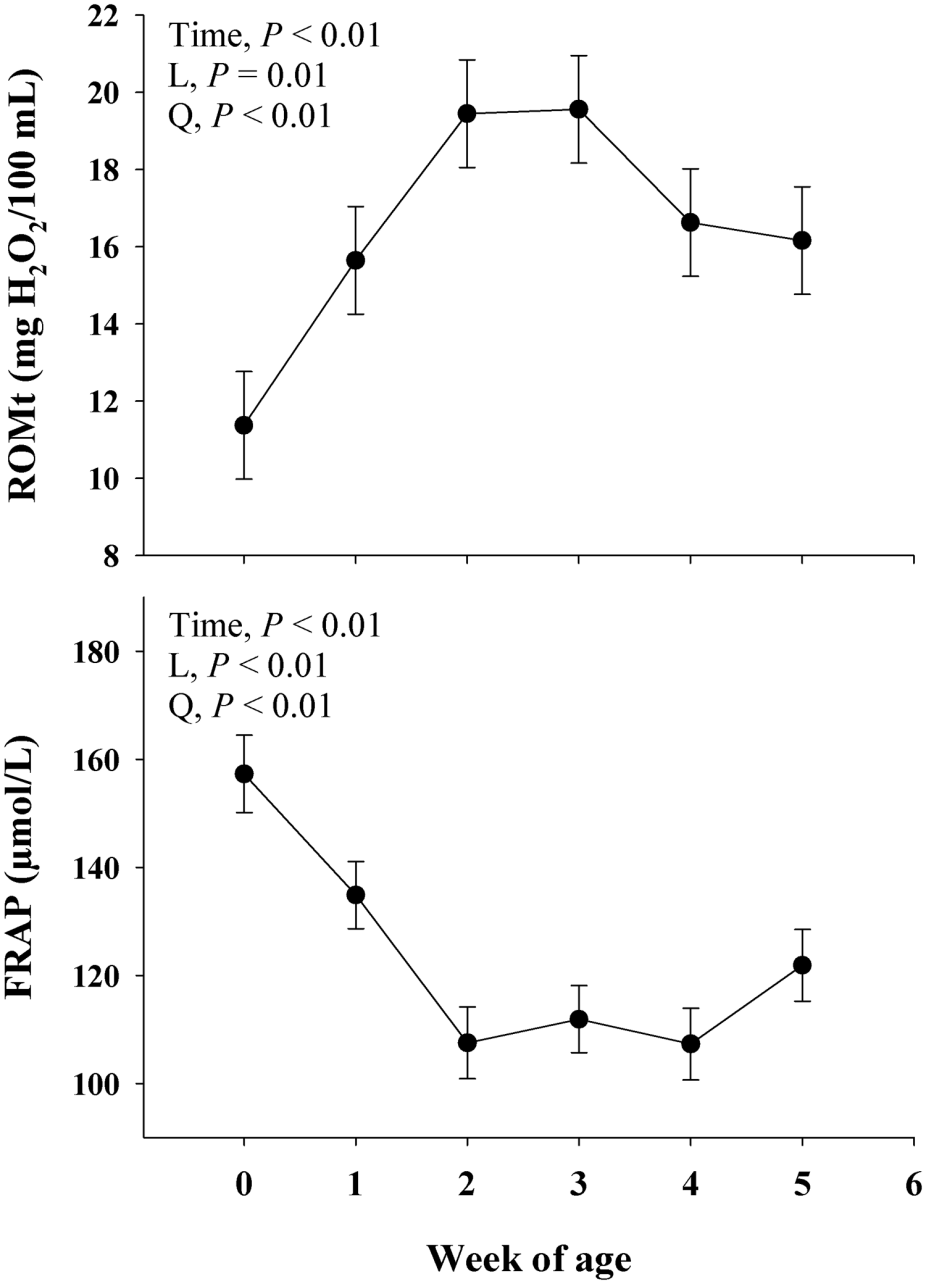
Profiles of oxidative stress [reactive oxygen metabolites (ROM) and ferric reducing antioxidant power (FRAP)] biomarkers in blood during the neonatal period in Jersey calves. The time effect (*P* < 0.10) were further analyzed through orthogonal contrast in order to determine linear (L) and quadratic (Q) effects over time. Values are means, with standard errors represented by vertical bars.

Main effect of time on inflammatory biomarkers are presented in Fig 5. There was a main effect (*P* < 0.01) on ROM and FRAP. There was a linear (*P* ≤ 0.01) and quadratic (*P*< 0.01) response on ROM and FRAP. A positive quadratic effect was observed for ROM, and a negative quadratic effect was observed for FRAP (Fig 5).

### Gene expression

#### Immune receptors and proinflammatory cytokines

**Fig 6:**
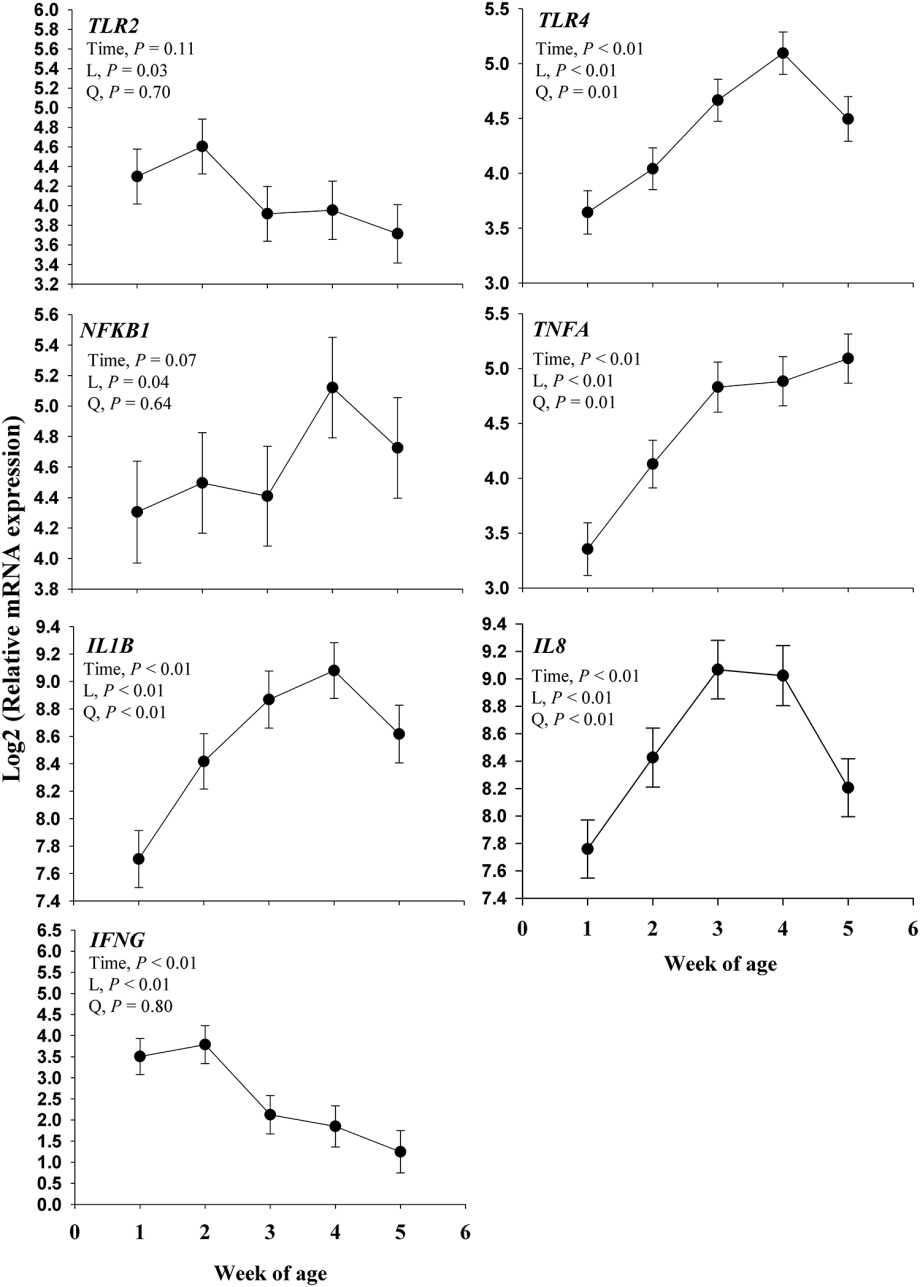
Expression of genes related to the activation of the innate immune system (*TLR2* and *TLR4*) and pro-inflammatory response (*NFKB1*, *TNFA*, *IL1B*, *IL8*, and *IFNG*) in fecal samples collected during the neonatal period of Jersey calves. The time effect (*P*< 0.10) were further analyzed through orthogonal contrast in order to determine linear (L) and quadratic (Q) effects over time. Values are means, with standard errors represented by vertical bars.

Main effects for immune receptors related genes are shown in Fig 6. There was a time effect (*P* <0.01) on *TLR4*, *TNFA*, *IL1B*, *IL8*, and *IFNG* and a trend on *NFKB1*(*P* = 0.07) over time. There was a linear response (*P*< 0.04) for all genes evaluated. A positive quadratic effect (*P* = 0.05) was observed for *TLR4*, *TNFA*, *IL1B*, and *IL8*. No quadratic effect (*P* ≥ 0.64) was observed for *TLR2*, *NFKB1* or *IFNG*, however, the expression of *TLR2* and *IFNG* decreased (*P* ≤ 0.03) over time while *NFKB1* increased (*P* = 0.04).

#### Cell membrane transporters

**Fig 7:**
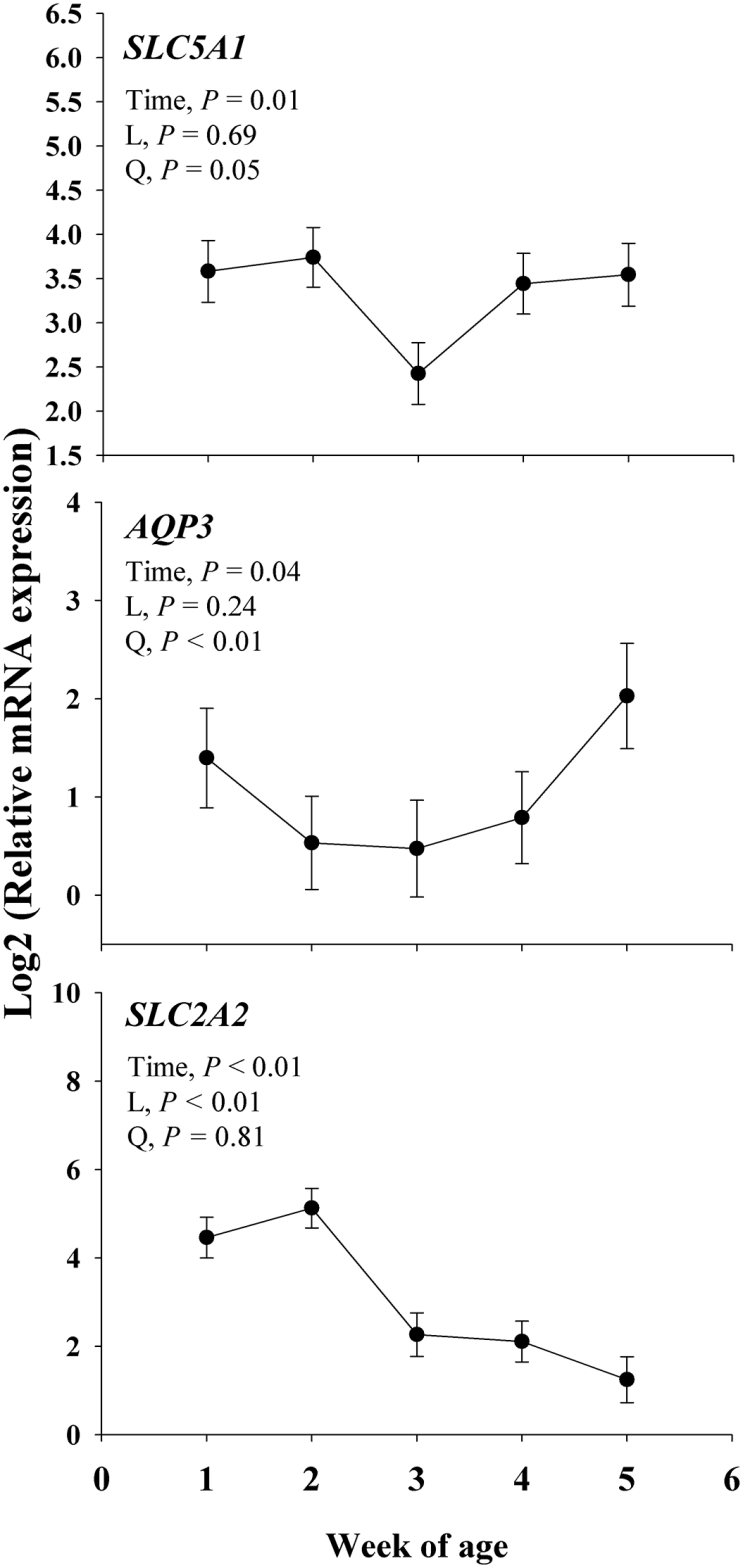
Expression of genes coding for proteins involved in ions (*SLC5A1*, *SLC2A2*) and water (*AQP3*) transporters, in fecal samples collected during the neonatal period of Jersey calves. The time effect (*P* < 0.10) were further analyzed through orthogonal contrast in order to determine linear (L) and quadratic (Q) effects over time. The *P* values for main effect of time are shown in each plot. Values are means, with standard errors represented by vertical bars.

Main effects for genes associated with cell membrane transporters are shown in Fig 7. There was a time effect (*P* < 0.04) observed for *SLC5A1*, *AQP3*, and *SLC2A2*. A linear response was observed (*P* < 0.01) for *SLC2A2* and with no linear response for *AQP3* and *SLC5A1*. A negative quadratic effect (*P* ≤ 0.05) was observed on *AQP3* and *SLC5A1*.

## Discussion

### Animal performance

Although there was an evident increase in scours during 2 and 3 wk (Fig 1B), overall growth was not compromised throughout the study. The starter intake at the end of the trial was 0.193 kg/d, which is in agreement with starter intakes (0.2 kg/d starter intake) reported by others [24]. Although ADG reached a nadir level at wk 2, it increased linearly until wk 5 (Fig 1E), and, in fact, the overall ADG (0.42 vs 0.21 kg/d) was greater than that reported in Jersey calves across treatments in [25].

### Blood biomarkers for energy and protein metabolism during early life

The concentration of BHB was likely increased linearly by the increased starter intake due to the stimulation of the ruminal fermentation, which is an indicator of rumen development of young ruminants [26]. The BHB levels in this study ranged from 0.12 mmol/L (wk 0) to 0.20 mmol/L (wk 5) which are comparable to those observed by Quigley et al. [26] and Klotz and Heitmann [25], where BHB concentrations increased in neonatal Jersey calves regardless of dietary treatments. The increased concentration of glucose throughout this trial can be associated with a physiological response, where neonates have to develop their gluconeogenesis pathway to generate endogenous energy to reach the normal metabolism [27]. In our study, glucose concentration ranged from 6.8 mmol/L at wk 0 to 7.5 mmol/L at wk 5, and was higher than other studies [25, 28].

In this study, the blood creatinine was in agreement with Khan et al. [29], reporting creatinine values from 80.4 (wk 2) to 67.2 μmol/L (wk 12) and in both experiments creatinine levels decreased with age. The latter suggests that creatinine levels at birth might be associated with a transfer from maternal creatinine as observed in humans [30]. The overall increase in blood urea in our study is associated with the development of the ruminal fermentation, which has been demonstrated that blood urea concentrations in dairy calves increase over time due to rumen development stimulated by solid feed, especially after weaning [31]. Also, plasma urea concentration increased in neonatal dairy calves, when calves began to consume greater amounts of grain [32, 33] which result in greater production of ruminal ammonia, and consequently more metabolized urea into the bloodstream.

### Biomarkers of liver function

Liver function is commonly depressed during inflammatory episodes [17], which results in reduced albumin, PON, and cholesterol in the blood. In contrast, greater blood GGT and GOT are associated with liver cell damage [34]. Therefore, in the current study, the linear increase of cholesterol, albumin, and PON in the blood of calves does not suggest a liver dysfunction even during 2 wk of age when the fecal score was maximal. In addition, the clearance of total bilirubin in blood is associated with a progressive increase in liver function (Fig 3). The high GGT concentration at wk 0 can be associated with colostrum intake, as this enzyme is present in high levels in colostrum (Gungor et al., 2004). Then, the decreased GGT concentration over time is in accordance with other studies in neonatal dairy calves [35, 36].

### Biomarkers of oxidative stress

Oxidative stress is driven by the imbalance between the production of reactive oxygen metabolites (ROM) and antioxidants. The process of phagocytosis by which leukocytes such as neutrophils and macrophages can destroy invading pathogens can also lead to increased production of ROM [37]. The fact that ROM reached a maximum level in blood at 2 wk of age (19 mg H_2_O_2_/100 mL), together with the decrease in FRAP concentration (antioxidant parameter; Fig 5) during the same time which calves experienced an increase in the fecal score. Taken these data together, suggest that this is a physiological response during a period of stress such as diarrhea. Therefore, increased ROM could be associated with the increase in diarrhea events leading to a potential increase phagocytosis, arachidonic acid metabolism, oxidation of tissue reserves, among other mechanisms to counteract this condition [38].

### Inflammatory response in fecal RNA and blood biomarkers

Toll-like receptors (TLRs) present in the cell membrane can identify an invading pathogen, and start an intracellular signaling pathway with the purpose to activate the transcription factor *NFKB1* which promotes the transcription of genes encoding proinflammatory proteins (e.g., cytokines and chemokines), such as *IL1B*, *TNFA*, *IL6* and *IL8* [39]. Among TLRs, TLR2 and TLR4 are perhaps the most widely studied, which recognize components of the bacterial cell walls such as lipoteichoic acids (LTA) and lipopolysaccharides (LPS) on Gram-positive and Gram-negative bacteria, respectively [40]. Therefore, it is plausible that the upregulation of *TLR4* mRNA expression observed in fecal samples was triggered by the presence of gram-negative bacteria (i.e., *E*. *coli*). Similar responses have been observed in other tissues such as the mammary gland where an upregulation of *TLR4* was observed in dairy cows experiencing an inflammatory condition due to LPS [41, 42] or *E*. *coli* challenge [43]. Among the limited data on *TLR4* expression in GI tract in dairy calves, Malmuthuge et al. [44] did not observe differences in *TLR4* expression across several GI tract tissues (i.e., rumen, jejunum, ileum, cecum, and colon) in 3 wk-old calves. Although an upregulation of *TLR4* was observed with a maximal expression at 4 wk, this does not precisely overlap with the increase in fecal scores at 1 wk. However, the latter could be associated with an alternative pathogen and consequently mechanism, other than TLR4 pathway, responsible for triggering the increased fecal scores at 1 wk. Certainly, there still much to know regarding the GI tract of neonatal calves, for instance, Ontsouka et al. [45] described the importance in the maturation of the GI tract in neonatal calves and how bioactive substance in colostrum can affect this maturation. Therefore, the latter could be another potential explanation for the delay observed in TLR4 with respect of the increased fecal scores in 1 wk observed in this study.

The concomitant downregulation over time observed in this study for *TLR2* and *IFNG* can be associated with early life exposure to *Mycobacterium* species in colostrum [46], for which TLR2 has been reported as the main TLR to recognized these pathogens [47], and IFNγ has a significant role during the activation of TLR2 signaling pathway [48].

Because of the downregulation of *TLR2*, it is likely that most of the inflammatory response observed in fecal RNA was originated through the upregulation of *TLR4* at 4 wk. For instance, the simultaneous upregulation of *NFKB1* in fecal RNA at 4 wk and its target genes *TNFA*, *IL1B*, and *IL8* can be associated with an overall activation of the TLR4 pathway. A similar effect was observed by Santos et al. [49], where ileal sections of calves were inoculated with *Salmonella typhimurium*, and the overall results demonstrated an upregulation of *IL8* and *IL1B*.

Serum cytokines such as IL-6 have been used as a pro-inflammatory [50, 51] or anti-inflammatory [52, 53] biomarker, and it is one of the main cytokines responsible for initiating the hepatic acute phase response (APR) [54]. During inflammation, IL-6 can be synthesized by the intestinal mucosa and enterocyte by which stimulates enterocytes production of +APP [55, 56]. Upon an inflammatory condition, the APR increases hepatic synthesis of positive acute phase proteins (+APP) such as SAA, ceruloplasmin, and haptoglobin, and the concomitant decrease in the production of negative acute phase proteins (-APP), such as albumin and apolipoproteins [34, 57]. The results of this study showed a simultaneous increase in IL-6 in blood and fecal scores, which indicates that calves underwent an inflammatory response. In fact, Fischer et al. [58] reported that serum IL-6 is a reliable biomarker for the inflammatory response to diarrhea in calves. The latter was further confirmed by increased concentrations of +APP such as ceruloplasmin, haptoglobin, and SAA which has been observed previously in neonatal dairy calves infected with *Salmonella* [56, 59, 60] and inoculated with bovine viral diarrhea virus [61]. As expected inoculation with *Salmonella* causes diarrhea resulting in a fecal score of 2.5 [60], which is in agreement with our study. Overall, in our study, the pattern of fecal score and pro-inflammatory blood biomarkers (peaking both between 2 and 3 wk of age) indicate a pro-inflammatory response during diarrhea in neonatal dairy calves. The inflammatory response observed in fecal RNA did not overlap with that observed in fecal scores and blood biomarkers at 2 and 3 wk. However, the fact that inflammation-related genes in fecal RNA followed a similar pattern as fecal scores and blood biomarkers of inflammation provide support for fecal RNA being a promising method to study intestinal response to inflammation and its variation over time in neonatal dairy calves. Future improvements to the fecal RNA method will consolidate its reliability to study gut health and response to inflammation, dietary conditions, and growth among others.

### Cell membrane transporters in fecal RNA

The absorption of glucose and galactose from the intestinal lumen into the enterocytes is regulated by the sodium-dependent glucose transporter SGLT1 (gene symbol *SLC5A1*) present in the apical membrane [62]. After glucose, galactose, and fructose are transported into the enterocytes, these are further translocated out of the cell into the portal venous system through the facilitated transporter GLUT2 present in the basolateral membrane [63]. Previous work in colonic human cells and rabbits showed a decrease in the mRNA expression of *SLC5A1* in response to an inflammatory condition induced by synthetic TNFα [64, 65]. Similarly, the downregulation of *SLC5A1* in fecal RNA can be partially related to the time of increased inflammatory response observed in blood biomarkers and fecal score. Thus, the decreased expression of *SLC5A1* around the same time of the diarrhea could be suggestive of a physiological response to an inflammatory condition.

As mentioned above GLUT2 is a major transporter of carbohydrates and is encoded by *SLC2A2*, which was detected in gut segments (i.e., duodenum and jejunum) of 4 d old dairy calves [66]. The hepatic mRNA expression of *SLC2A2* was downregulated overtime in transition calves from preruminant to ruminants [67], indicating a decreased in the flux of glucose in the portal vein. Therefore, the downregulation overtime in *SLC2A2* expression in the fecal samples can be associated with the transition from preruminant to ruminant in those calves, where *SLC2A2* expression decreased as the rumen functionality increased.

Another family of cell membrane transporters is the aquaporins, and the Aquaporin-3 (encoded by *AQP3*) transports water, glycerol, urea, and other small uncharged solutes across the plasma membrane [68, 69]. Previous studies have shown that *AQP3* is down-regulated during inflammatory bowel disease in humans [70] and rats [71]. Indeed, it was reported that during bacterial-induced diarrhea in mice the flux of water through the enterocytes is altered compared to a healthy gut environment, where water flux in bacterial-induced diarrhea is blocked from entering or exiting the lumen of the intestine into the epithelium, mainly due to the mislocalization of Aquaporin-3 within the enterocytes [72]. Thus, the reduced *AQP3* expression observed in the fecal RNA during diarrhea in the present study might be an indication of restricted water exchange between the intestinal lumen and epithelium as a physiological response to diarrhea.

## Conclusions

The greater concentration of biomarkers of inflammation in the blood coupled with the upregulation of inflammatory-related genes in fecal RNA from neonatal dairy calves undergoing a mild diarrhea is indicative of an inflammatory response. Such a response confirmed our hypothesis, where physiological changes such as inflammation in response to diarrhea could be detected through transcriptional alterations in fresh fecal samples from neonatal dairy calves. Although further optimization to this method is required in the future, results from this study suggest that RNA isolated from fecal samples has a potential to be a reliable tool to evaluate physiological changes not only due to diarrhea but also to other factors such as dietary treatments.

## Supporting information

S1 Table Gene ID, GenBank accession number, gene symbol, hybridization position, sequence and amplicon size of primers used to analyze gene expression. (PDF)

S2 Table Sequencing results of PCR products from primers of genes used for this experiment. (PDF)

S3 Table Sequencing results of genes using BLASTN (http://www.ncbi.mln.nih.gov) from NCBI against nucleotide collection. (PDF)

S1 Fig Distribution of calves with a given diarrhea over time. (PDF)

S1 File Description of protocols for RNA isolation, cDNA synthesis, design and evaluation of primers, and polymerase chain reaction (PCR). (PDF)
