# Supplementary material for: Transcriptional changes detected in fecal RNA of neonatal dairy calves undergoing a mild diarrhea are associated with inflammatory biomarkers
Source: PLoS One. 2018 Jan 26;13(1):e0191599. doi: 10.1371/journal.pone.0191599 (PMC5786293; doi:10.1371/journal.pone.0191599)
Supplement: S1 Table — (PDF) [file pone.0191599.s001.pdf]

S1 Table. Gene ID, GenBank accession number, gene symbol, hybridization position, sequence and amplicon size of primers used to analyze gene expression.

Gene ID	Accession #	Gene	Primers	Primers (5'-3')	bp
281418	XM_010804358.2	*PPIA*	F.125	TCTGAGCACTGGAGAGAAAGGATTTG	88
			R.212	GAAGTCACCACCCTGGCACATA	
282361	XM_005218131.3	*SLC5A1*	F.1292	GCAGGACGGTTGTTC	107
			R.1398	GTGATGGACTGGATGTAG	
282357	NM_001103222	SLC2A2	F.1427	TTATGTGTTTTTCCTTTTGCTG	82
			R.1508	ATTTTCCTTTGGTTTCTGGAAC	
780866	NM_001079794	*AQP3*	F.398	GGGTTGTATTACGATGCGATCTG	81
			R.478	AAAGATGCCAGCTGTGCCATTG	
