# Supplementary material for: Transcriptional changes detected in fecal RNA of neonatal dairy calves undergoing a mild diarrhea are associated with inflammatory biomarkers
Source: PLoS One. 2018 Jan 26;13(1):e0191599. doi: 10.1371/journal.pone.0191599 (PMC5786293; doi:10.1371/journal.pone.0191599)
Supplement: S2 Table — (PDF) [file pone.0191599.s002.pdf]

S2 Table. Sequencing results of PCR products from primers of genes used for this experiment.

Gene	Sequence
PPIA	GTCTGCTTTAGAATAATTCCGGGATTTATGTGCCAGGGTGGTGACTGAAG
SLC5A1	GCATCGCTGGGTGCCATCGTGCAGTCAGCACAGAGTGGACAGCTCTTCGACTACATCCA GTCCATCAAG
AQP3	GCATAACAGCTTATTGTTTCGGGCCCATGGCACAGCTGGCATCTTATA
SLC2A2	CTTCTGCTTTTTTAAGTTCCAGAACCAAAGGAAAATA
