# Supplementary material for: Transcriptional changes detected in fecal RNA of neonatal dairy calves undergoing a mild diarrhea are associated with inflammatory biomarkers
Source: PLoS One. 2018 Jan 26;13(1):e0191599. doi: 10.1371/journal.pone.0191599 (PMC5786293; doi:10.1371/journal.pone.0191599)
Supplement: S3 Table — (PDF) [file pone.0191599.s003.pdf]

S3 Table. Sequencing results of genes using BLASTN (http://www.ncbi.mln.nih.gov) from NCBI against nucleotide collection

Accession #	Name	Best hit in NCBI	Coverage	Identity
XM_010804358.2	*PPIA*	PREDICTED: Bos taurus peptidylprolyl isomerase A (PPIA), transcript variant X2, mRNA	74%	100%
XM_005218131.3	*SLC5A1*	PREDICTED: Bos taurus solute carrier family 5 (sodium/glucose cotransporter), member 1 (SLC5A1), transcript variant X1, mRNA	97%	97%
NM_001079794.1	*AQP3*	Bos taurus aquaporin 3 (Gill blood group) (AQP3), mRNA	78%	95%
XM_005201668.3	*SLC2A2*	PREDICTED: Bos taurus solute carrier family 2 (facilitated glucose transporter), member 2 (SLC2A2), transcript variant X1, mRNA	54%	93%
