# Supplementary material for: Transcriptional changes detected in fecal RNA of neonatal dairy calves undergoing a mild diarrhea are associated with inflammatory biomarkers
Source: PLoS One. 2018 Jan 26;13(1):e0191599. doi: 10.1371/journal.pone.0191599 (PMC5786293; doi:10.1371/journal.pone.0191599)
Supplement: S1 Fig — (PDF) [file pone.0191599.s004.pdf]

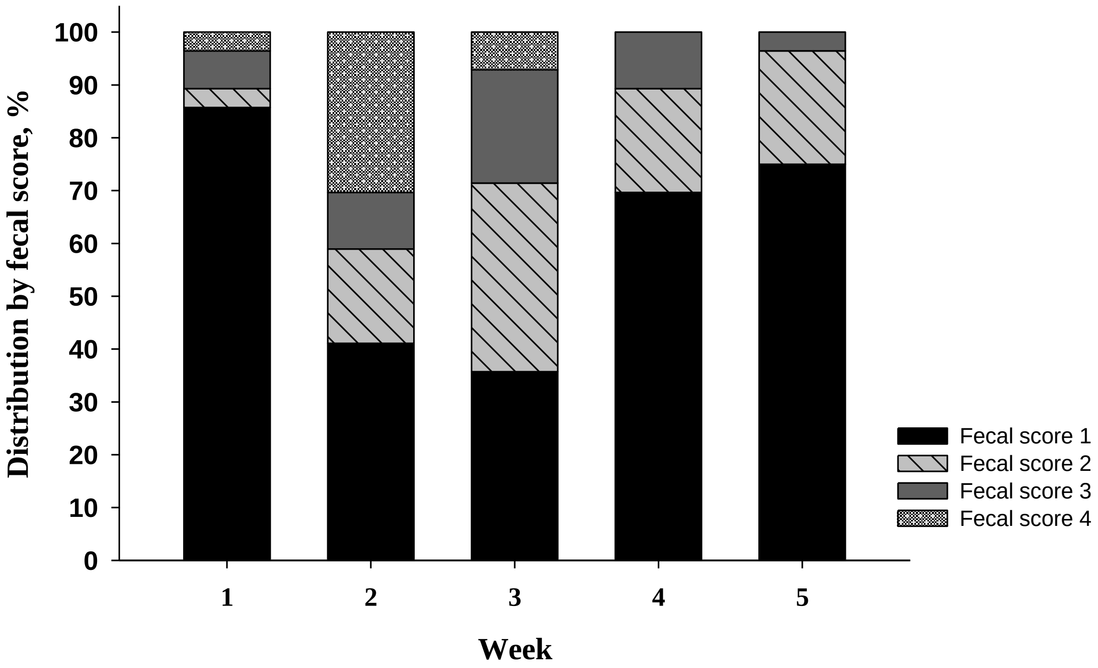


**Figure S1.** Descriptive representation of daily fecal score distribution per week in neonatal Jersey calves.
