# Supplementary material for: Transcriptional changes detected in fecal RNA of neonatal dairy calves undergoing a mild diarrhea are associated with inflammatory biomarkers
Source: PLoS One. 2018 Jan 26;13(1):e0191599. doi: 10.1371/journal.pone.0191599 (PMC5786293; doi:10.1371/journal.pone.0191599)
Supplement: S1 File — (PDF) [file pone.0191599.s005.pdf]

S1 File.

RNA isolation and cDNA synthesis

RNA isolation from fecal samples was performance using the RNeasyPlus Mini Kit (Qiagen, Cat. No. 74134), following the manufacturer's instructions with some modifications. Briefly, the fecal samples (~200 mg) were weighed and placed into a 2 mL RNase/DNase free tube (Metal Bead beating tubes, Cat# 13117-50, Qiagen) containing 1 bead (Qiagen; Cat. No: 69989, 5 mm) and 1.2 mL of Trizol (Invitrogen Corp., Carlsbad, CA) at 4 °C were added. Then, the samples were homogenized twice for 30 s with 1 min incubation time on ice using a Bullet Blender Next Advance (Laboratory Instruments, USA). After homogenization, the lysate was transferred to a 1.7 mL microtube and 240 µL of chloroform (EMD Millipore, Germany, Cat. No. CX1055-6) at 4 °C was added in order to isolate the RNA from the organic phase. The tubes were mixed well by hand for 15 s and followed by an incubation at 4 °C for 10 min. After centrifugation at 13,000 x *g* for 15 min at 4 °C, the upper phase supernatant was transferred in a new 1.7 mL microtube. Then, the Total RNA isolation was performed following the procedure recommended by Qiagen (RNeasyPlus Mini Kit; Cat. No. 74134). The final RNA was collected in 50 µL RNase-free water. The RNA was quantified using SpectraMax® Plus 384(Molecular Devices, Sunnyvale, CA, USA) and the quality (RNA integrity – RIN) was evaluated using a 2100 Bioanalyzer Instruments (Agilent, USA).

Primer design and Evaluation

Prior to qPCR, primers were verified through a 20 µL PCR reaction, composed of 1 µL each of forward and reverse primers, 10 µL of 1×SYBR Green master mix (Cat# 4367659, Applied Biosystems, Woolston Warrington, UK) and 8 µL of cDNA (a pool cDNA amplified from all RNA samples was utilized to ensure the identification of genes), following the conditions: 2 min at 50°C, 10 min at 95°C, and 40 cycles with 15s at 95°C followed by 1 min at 60°C. Five microliters of each PCR product was run in 2% agarose gel stained with EZ-vision® (Cat# N391-15MLDRP, Amresco, Solon, OH), and the remaining 15 µL were cleaned with DNA clean & Concentrator Kit (Cat# 11-302C, Zymo Research, USA) and sequenced at the Center for Genome Research and Biocomputing (CGRB) at Oregon State University.

qPCR

qPCR was performed in a MicroAmp Optical 384-Well Reaction Plate (Cat# 4309849 , Applied Biosystems, Grand Island, NY). Within each well, 4µL of diluted cDNA combined with 6µL of mixture composed of 5 µL 1×SYBR Green master mix (Cat# 4367659, Applied Biosystems, Woolston Warrington, UK), 0.4µL each of 10µM forward and reverse primers, and 0.2 µL of DNase/RNase-free water. Three replicates were run for each sample and a nontemplate control (NTC) was run for each gene analyzed. qPCR was conducted in ABI Prims 7900 HT SDS instrument (Applied Biosystems) following the conditions: 2 min at 50°C, 10 min at 95°C, and 40 cycles with 15s at 95°C followed by 1 min at 60°C. A dissociation curve was performed (gradient from 95C to 60°C to 95°C) to check for amplicon quality.
